# Visualization of Relative Measures of Association: Points and Error Bars With an Appropriate Axis Scale

**DOI:** 10.2188/jea.JE20230052

**Published:** 2023-09-05

**Authors:** Ryosuke Fujii

**Affiliations:** 1 Institute for Biomedicine (affiliated to the University of Lübeck), Eurac Research, Bolzano/Bozen, Italy; 2 Department of Preventive Medical Science, Fujita Health University School of Medical Sciences, Aichi, Japan; 3 Department of Preventive Medicine, Nagoya University Graduate School of Medicine, Nagoya, Japan

In biomedical studies, relative measures (eg, odds ratio, hazard ratio, rate ratio, and risk ratio [RR]) are often used to evaluate associations between exposures and outcomes with graphical presentation. Although cautious points for graphical presentation of these indicators have been discussed,^1^ there are still some inappropriate graphical presentations. This letter summarizes the current situation with a brief literature review and provides an extended discussion referring to past publications on this issue.

We reviewed the original articles published in the following four epidemiological journals between April 2022 and March 2023: the *American Journal of Epidemiology*, the *European Journal of Epidemiology*, the *International Journal of Epidemiology*, and the *Journal of Epidemiology* (Table 1). A total of 102 articles presented at least one figure for relative measures of association. We categorized these articles as “Appropriate” and “Inappropriate”, followed by further dividing into “Axis starting from 0”, “Axis with an arithmetic scale”, and “Undetermined” for the inappropriate category. Of the 102 articles, 50 (49.0%) articles failed to show appropriate graphs and 49 (48.0%) faced an issue with the axis scale. The worst case, presenting bar graphs starting from 0 with an arithmetic scale, was observed in three articles (2.9%). The lists of papers and its category for each journal are provided in eTable 1, eTable 2, eTable 3, and eTable 4. The percentage of papers with issues with the axis scale was still high compared to that reported in 2010.^2^ This problem is prone to mislead not only academic readers but also the general public, who are not familiar with the interpretation of these indices. Therefore, we need to carefully display these types of results in figures.

**Table 1.** Summary of literature review for data visualization of relative measures in four journals in the field of epidemiology (April 2022–March 2023)^a,b^

Major journals in the field of epidemiology Publication frequency	Inappropriate	Axis with an arithmetic scale^c^	Undetermined^c,d^	Total	Appropriate	*N* of papers reporting relative measures in Figure^e^	*N* of papers reporting relative measures in Table^e^
	Axis starting from 0^c^						
The American Journal of Epidemiology, Monthly	2	12	0	12 (48.0%)	13 (52.0%)	25	45
The European Journal of Epidemiology, Monthly	0	13	0	13 (44.8%)	16 (55.2%)	29	27
The International Journal of Epidemiology, Bimonthly	0	16	0	16 (42.1%)	22 (57.9%)	38	49
The Journal of Epidemiology, Monthly	1	8	1	9 (90.0%)	1 (10.0%)	10	30

^a^If an article presented Figures in both appropriate and inappropriate ways, it was categorized as “inappropriate”.

^b^In this literature review, there was no example like Figure 1C (non-transparent bar + error bars with a logarithmic scale).

^c^If an article has multiple issues, each issue is counted up.

^d^“Undetermined” includes problems for a strange scale for relative measures.

^e^If an article reported both table and figure, each type is counted up.

To improve the clarity of figures, this letter revisits past discussions once again and explains the process and rationale for appropriate visualization. Here, Figure 1 was created by adopting the figure presented by Szklo and Nieto in their book. The first point to be considered is the reference value.^1^^,^^3^ Figure 1A and Figure 1B present less favorable examples of bar charts starting from 0 or 1. The bar chart is suitable when counting the number of data and starts from 0. However, relative measures are calculated with 1 as a reference value, thus bar charts beginning with 0 are out of place (at least they should start from 1). In fact, Figure 1A gives an incorrect impression that a RR of 2.0 is a fourth larger than a RR of 0.5. The second point is an axis scale. As mentioned above, the axis scale is still a major issue, even though this is the most discussed part in the past. Figure 1B and Figure 1C compare the axis with arithmetic and a log-transformed scales. Obviously, in Figure 1C with a logarithmic scale, you can interpret that a RR of 2.0 is the same magnitude (different direction) as a RR of 0.5. However, in Figure 1B with an arithmetic scale, this is also likely to be misinterpreted that a RR of 2.0 is twice as large as a RR of 0.5. To avoid misleading, it is preferable to transform with a logarithmic scale or reciprocal scale. Finally, Figure 1C and Figure 1D compare different visual channels. In Figure 1C, either error bar is sometimes indistinct using bar charts filled in dark colors. It is impossible to determine a range of confidence intervals at a glance. Therefore, if bars can be made transparent, it would be acceptable. Meanwhile, it is visible in the case of using points and errors (Figure 1D).^2^^,^^4^ This improvement is not substantial compared with previous issues, but it could facilitate a better discussion based on confidence intervals rather than a discussion focusing on only point estimates and *P*-values. This graphical presentation using points and error bars has commonly appeared in figures for meta-analysis.

**Figure 1.**
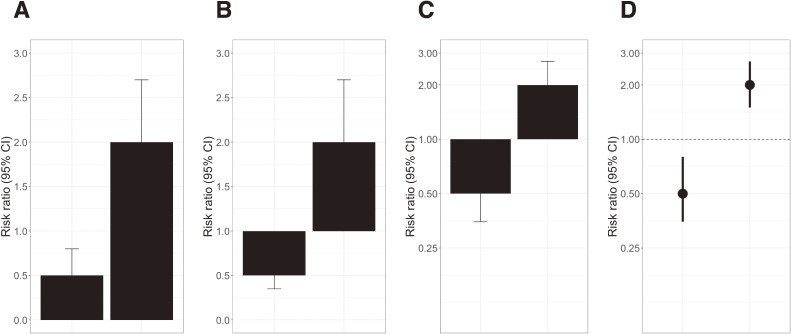
Different visualization patterns for risk ratio. (A) Bar charts beginning at 0 with an arithmetic scale; (B) Bar charts beginning at 1 with an arithmetic scale; (C) Bar charts beginning at 1 with a logarithmic scale; (D) Points and error bars with a logarithmic scale. There are no cases like Figure 1C in the current literature review. (Szklo, M., & Nieto, F. J., Epidemiology: Beyond the basics, 2006: Jones & Bartlett Learning, Burlington, MA. www.jblearning.com. Reprinted with permission). CI, confidence interval.

For clear and precise data visualization of point estimates for relative measures, it is preferable to draw them as point + error bars with a logarithmic scale or transparent bar chart + error bars starting from 1 with a logarithmic scale. Researchers need to pay attention to this issue not only in papers and conference presentations but also in dissemination to the public. As a final note, remember that there is a trade-off between visualizing the results in figures and showing them as tables; in other words, it is often impossible to correctly reproduce numerical results from figures. The R codes of data visualization in relative measures for more practical examples are available at https://github.com/fujichaaan/je_point_estimates_rm.

## SUPPLEMENTARY MATERIAL

eTable 1. Publication list for four different categories in Table 1 (*Am J Epidemiol*, n = 25)

eTable 2. Publication list for four different categories in Table 1 (*Eur J Epidemiol*, n = 29)

eTable 3. Publication list for four different categories in Table 1 (*Int J Epidemiol*, n = 38)

eTable 4. Publication list for four different categories in Table 1 (*J Epidemiol*, n = 10)
