# Supplementary material for: Visualization of Relative Measures of Association: Points and Error Bars With an Appropriate Axis Scale
Source: J Epidemiol. 2023 Sep 5;33(9):487–8. doi: 10.2188/jea.JE20230052 (PMC10409530; doi:10.2188/jea.JE20230052)
Supplement: Supplementary file 1 [file je-33-487-s001.pdf]

eTable 1. Publication list for four different categories in Table 1 (*Am J Epidemiol*, n=25)

Axis starting from 0	Axis with arithmetic scale	Undetermined	Appropriate
https://academic.oup.com/aje/article/191/5/874/6497582	https://academic.oup.com/aje/article/192/2/205/6747774		https://academic.oup.com/aje/article/192/1/41/6667512
https://academic.oup.com/aje/article/191/10/1732/6623870	https://academic.oup.com/aje/article/192/2/182/6765226		https://academic.oup.com/aje/article/192/2/158/6765228
	https://academic.oup.com/aje/article/192/2/230/6759399		https://academic.oup.com/aje/article/192/2/171/6776164
	https://academic.oup.com/aje/article/191/4/613/6446894		https://academic.oup.com/aje/article/192/3/408/6901498
	https://academic.oup.com/aje/article/191/4/665/6438539		https://academic.oup.com/aje/article/191/4/570/6497583
	https://academic.oup.com/aje/article/191/5/874/6497582		https://academic.oup.com/aje/article/191/5/843/6397977
	https://academic.oup.com/aje/article/191/7/1224/6541606		https://academic.oup.com/aje/article/191/6/987/6528789
	https://academic.oup.com/aje/article/191/10/1677/6553935		https://academic.oup.com/aje/article/191/6/1040/6507339
	https://academic.oup.com/aje/article/191/11/1897/6653176		https://academic.oup.com/aje/article/191/6/1081/6511805
	https://academic.oup.com/aje/article/191/10/1732/6623870		https://academic.oup.com/aje/article/191/7/1235/6542790
	https://academic.oup.com/aje/article/191/12/2002/6653178		https://academic.oup.com/aje/article/191/8/1383/6511811
	https://academic.oup.com/aje/article/191/12/2037/6673027		https://academic.oup.com/aje/article/191/12/1990/6623871
			https://academic.oup.com/aje/article/191/12/2014/6656957

Dark shaded cells are duplicated somewhere.

eTable 2. Publication list for four different categories in Table 1 (*Eur J Epidemiol*, n=29)

Axis starting from 0	Axis with arithmetic scale	Undetermined	Appropriate
	https://link.springer.com/article/10.1007/s10654-021-00835-4		https://link.springer.com/article/10.1007/s10654-022-00858-5
	https://link.springer.com/article/10.1007/s10654-022-00873-6		https://link.springer.com/article/10.1007/s10654-022-00923-z
	https://link.springer.com/article/10.1007/s10654-022-00863-8		https://link.springer.com/article/10.1007/s10654-022-00851-y
	https://link.springer.com/article/10.1007/s10654-022-00886-1		https://link.springer.com/article/10.1007/s10654-022-00881-6
	https://link.springer.com/article/10.1007/s10654-022-00850-z		https://link.springer.com/article/10.1007/s10654-022-00856-7
	https://link.springer.com/article/10.1007/s10654-022-00900-6		https://link.springer.com/article/10.1007/s10654-022-00842-z
	https://link.springer.com/article/10.1007/s10654-022-00898-x		https://link.springer.com/article/10.1007/s10654-021-00763-3
	https://link.springer.com/article/10.1007/s10654-022-00906-0		https://link.springer.com/article/10.1007/s10654-022-00868-3
	https://link.springer.com/article/10.1007/s10654-022-00905-1		https://link.springer.com/article/10.1007/s10654-022-00866-5
	https://link.springer.com/article/10.1007/s10654-022-00922-0		https://link.springer.com/article/10.1007/s10654-022-00899-w
	https://link.springer.com/article/10.1007/s10654-022-00945-7		https://link.springer.com/article/10.1007/s10654-022-00903-3
	https://link.springer.com/article/10.1007/s10654-022-00952-8		https://link.springer.com/article/10.1007/s10654-022-00897-y
	https://link.springer.com/article/10.1007/s10654-022-00891-4		https://link.springer.com/article/10.1007/s10654-022-00926-w
			https://link.springer.com/article/10.1007/s10654-022-00921-1
			https://link.springer.com/article/10.1007/s10654-022-00914-0
			https://link.springer.com/article/10.1007/s10654-022-00962-6

eTable 3. Publication list for four different categories in Table 1 (*Int J Epidemiol*, n=38)

Axis starting from 0	Axis with arithmetic scale	Undetermined	Appropriate
	https://academic.oup.com/ije/article/52/1/22/6770060		https://academic.oup.com/ije/article/52/1/71/6609600
	https://academic.oup.com/ije/article/52/1/44/6874795		https://academic.oup.com/ije/article/52/1/132/6589377
	https://academic.oup.com/ije/article/52/1/58/6645010		https://academic.oup.com/ije/article/52/1/190/6711464
	https://academic.oup.com/ije/article/52/1/96/6901321		https://academic.oup.com/ije/article/51/2/479/6321178
	https://academic.oup.com/ije/article/51/2/440/6511683		https://academic.oup.com/ije/article/51/2/567/6472565
	https://academic.oup.com/ije/article/51/3/709/6519493		https://academic.oup.com/ije/article/51/3/769/6370078
	https://academic.oup.com/ije/article/51/3/727/6561291		https://academic.oup.com/ije/article/51/3/789/6373963
	https://academic.oup.com/ije/article/51/3/885/6521336		https://academic.oup.com/ije/article/51/3/817/6513932
	https://academic.oup.com/ije/article/51/4/1219/6548226		https://academic.oup.com/ije/article/51/3/839/6454072
	https://academic.oup.com/ije/article/51/4/1230/6574395		https://academic.oup.com/ije/article/51/3/898/6348050
	https://academic.oup.com/ije/article/51/4/1243/6531916		https://academic.oup.com/ije/article/51/4/1120/6460628
	https://academic.oup.com/ije/article/51/5/1371/6617968		https://academic.oup.com/ije/article/51/4/1153/6548954
	https://academic.oup.com/ije/article/51/6/1733/6659906		https://academic.oup.com/ije/article/51/4/1178/6537509
	https://academic.oup.com/ije/article/51/6/1745/6665821		https://academic.oup.com/ije/article/51/4/1204/6537508
	https://academic.oup.com/ije/article/51/6/1847/6726398		https://academic.oup.com/ije/article/51/5/1396/6573377
	https://academic.oup.com/ije/article/51/6/1862/6659907		https://academic.oup.com/ije/article/51/5/1421/6554034
			https://academic.oup.com/ije/article/51/5/1469/6586602
			https://academic.oup.com/ije/article/51/5/1489/6550388
			https://academic.oup.com/ije/article/51/5/1568/6535585
			https://academic.oup.com/ije/article/51/6/1711/6731613
			https://academic.oup.com/ije/article/51/6/1775/6368705
			https://academic.oup.com/ije/article/51/6/1874/6599228

eTable 4. Publication list for four different categories in Table 1 (*J Epidemiol*, n=10)

Axis starting from 0	Axis with arithmetic scale	Undetermined	Appropriate
https://www.jstage.jst.go.jp/article/jea/32/5/32_JE20200384/_pdf/-char/en	https://www.jstage.jst.go.jp/article/jea/32/9/32_JE20200502/_pdf/-char/en	https://www.jstage.jst.go.jp/article/jea/33/2/33_JE20210099/_pdf/-char/en	https://www.jstage.jst.go.jp/article/jea/32/7/32_JE20200422/_pdf/-char/en
	https://www.jstage.jst.go.jp/article/jea/33/3/33_JE20210242/_pdf/-char/en		
	https://www.jstage.jst.go.jp/article/jea/33/3/33_JE20210052/_pdf/-char/en		
	https://www.jstage.jst.go.jp/article/jea/33/1/33_JE20200531/_pdf/-char/en		
	https://www.jstage.jst.go.jp/article/jea/32/7/32_JE20200538/_pdf/-char/en		
	https://www.jstage.jst.go.jp/article/jea/32/5/32_JE20200384/_pdf/-char/en		
	https://www.jstage.jst.go.jp/article/jea/32/5/32_JE20200305/_pdf/-char/en		
	https://www.jstage.jst.go.jp/article/jea/32/4/32_JE20210268/_pdf/-char/en		

Dark shaded cells are duplicated somewhere.
